# Early Integration of Palliative Care in the Care of Women with Advanced Epithelial Ovarian Cancer: The Time Is Now

**DOI:** 10.3389/fonc.2016.00083

**Published:** 2016-04-11

**Authors:** Linda Rosenbaum Duska

**Affiliations:** ^1^Division of Gynecologic Oncology, Department of Obstetrics and Gynecology, University of Virginia, Charlottesville, VA, USA

**Keywords:** ovarian cancer, palliative care, hospice, end of life, integration

Despite tremendous advances in surgery, primary chemotherapy, and novel treatments for recurrent disease, the diagnosis of advanced epithelial ovarian cancer in 2016 remains ultimately fatal in the majority of cases. Additionally, both the disease and the associated adjuvant treatment are not without substantial effect on overall quality of life. The cancer causes symptoms, but the treatment can cause even more significant problems, including neuropathy, nausea, fatigue, anorexia, and pain, among others. As oncology providers, we have a natural tendency to focus on the cancer and response to treatment rather than on the suffering of our patients related to treatment. Our patients in turn are reluctant to report their symptoms for fear that we will stop or change their treatment. As a result, though the cancer may be temporarily beaten into submission by aggressive surgery and adjuvant therapy, the patient may be simultaneously suffering from treatment-related symptoms that in some cases are permanent.

The early integration of palliative care in the treatment of women with advanced epithelial ovarian cancer allows us to address this quandary and not only improve quality of life but in some cases also prolong life. In the most well known of the randomized studies in cancer patients, 151 patients with newly diagnosed metastatic non-small cell lung cancer were randomized to integration of outpatient palliative care from the time of cancer diagnosis versus usual oncologic care (1). The early palliative care integration group not only had significant improvements in quality of life and mood but also (unexpectedly) had a statistically significantly improved overall survival (11.6 versus 8.9 months, *p* = 0.02), despite less aggressive intervention at the end of life. The results of this study have been confirmed by other studies in oncology patients (2–8). Our own Society of Gynecologic Oncology has advocated for inclusion of palliative care in the care of women with gynecologic cancer in their Choosing Wisely campaign.

What exactly is palliative care and how is this care best provided? The World Health Organization (WHO) defines palliative care as “an approach that improves the quality of life of patients and their families facing the problems associated with life-threatening illness, through the prevention and relief of suffering by means of early identification and impeccable assessment and treatment of pain and other problems, physical, psychosocial and spiritual”.^1^ Said another way, the palliative care approach to the patient is a holistic one that encompasses all aspects of the person and her caregivers/family, including those that many oncologists are ill equipped to address. Palliative care services are divided into primary palliative care and specialty primary care services. Most oncologists are trained to provide and feel comfortable providing primary palliative care for the patients; in the case of gynecologic oncologists, these services include basic symptom management and aligning treatment choices with patient goals. By contrast, specialty palliative care is provided by a team of providers, including a palliative care trained physician, nurse or advanced practice provider, social worker, chaplain, pharmacists, nutritionists, rehabilitation therapists, and direct care workers, among others. The specialty palliative care team aims to address all of the domains of palliative care, including not only the physical but also the emotional, spiritual, and social domains of care. A complete assessment of palliative care needs includes all of these domains and so requires a multidisciplinary approach to the patient and her family. Thus, the early integration of palliative care allows us as oncology providers to continue to care for our patients with ovarian cancer while also addressing their suffering and improving overall quality of life.

Why then have we not embraced the early integration of palliative care into the care of women with advanced ovarian cancer? The most important barrier to early integration is a lack of understanding about exactly what the term “palliative care” means (9). Both patients and providers mistakenly consider palliative care to be synonymous with end-of-life care and, thus, incompatible with anticancer therapy. Patients are concerned that the introduction of palliative care means that the oncologist is “giving up,” and data from providers have shown that many mistakenly consider palliative care to be synonymous with end-of-life care and, thus, incompatible with anticancer therapy (9, 10). The Institute of Medicine report “Dying in America” concluded that “one of the greatest remaining challenges is the need for better understanding of the role of palliative care among both the public and professionals across the continuum of care so that hospice and palliative care can achieve their full potential for patients and their families”.^2^ Said Misconceptions about the role of hospice and the hospice benefit are also prevalent, resulting in few patients with ovarian cancer taking advantage of the hospice benefit at the end of life and very late hospice referral (11).

There are other important barriers to the integration of palliative care early in the course of a malignancy, such as advanced ovarian cancer. These include the lack of availability of outpatient specialty palliative care services, poor reimbursement for palliative services, and a perceived lack of training and exposure by oncologists in provision of basic palliative care services. While most NCI-designated cancer centers have access to outpatient specialty palliative care services, these services are much less common in the community setting (12). Reimbursement for palliative care services remains poor, contributing to the lack of availability. Until recently, providers were not reimbursed for discussing advance care planning with their patients, a discussion that when done well can take a lot of time from a busy oncology clinic. Finally, surveys of both medical and gynecologic oncology fellows suggest that the trainees feel ill prepared to provide primary palliative care, to have difficult conversations, and to discuss end-of-life planning with their patients (13, 14). There is clearly room for improving the palliative curriculum and exposure in gynecologic oncology fellowships.

How then can we accomplish the early integration of palliative care into the care of our patients with advanced epithelial ovarian cancer? The first step should be improved and continuing education of both the public and health care providers regarding what services palliative care provides and regarding the value of these services. It may be as simple as re-naming palliative care to supportive care in some cases while we further the education effort to avoid the confusion of palliative care with end-of-life care (15, 16). Early referral should be prioritized in the setting of a disease like advanced ovarian cancer, as patients will gain the most benefit from this approach (17). We also need to focus on better education of our trainees regarding palliative care and end-of-life care, and we need to lobby for appropriate reimbursement for these time-intensive services. Finally, palliative care services will help our patients but will also help us as oncology providers by their ability to “share the load” (9).

Our patients with advanced ovarian cancer deserve the best overall care we can provide to them and to their families and caregivers. This best care includes the most aggressive surgery required to achieve complete cyto-reduction, the most effective chemotherapy (with clinical trial options), and the most appropriate and modern management of the inevitable disease recurrence; critical skills that all gynecologic oncologists learn and then master during their careers. But we must also be mindful of the important contribution of the early integration of palliative care services to our patients’ overall well-being. While the oncologist holds the “keys to the chemo,” resulting in a patient less likely to vocalize debilitating symptoms or non-medical concerns, the palliative care team is able to focus on other aspects of the patient and her family’s care. Our ultimate goal should not be only to improve overall survival, but also to improve overall survival in the context of improved quality of life in all domains.

## Author Contributions

The author confirms being the sole contributor of this work and approved it for publication.

## Conflict of Interest Statement

The author declares that the research was conducted in the absence of any commercial or financial relationships that could be construed as a potential conflict of interest.

## Footnotes

^1^http://www.who.int/cancer/palliative/definition/en/ Accessed Jan 2016

^2^https://iom.nationalacademies.org/~/media/Files/Report%20Files/2014/EOL/Report%20Brief.pdf Accessed Jan 2016
